# Advancing Simulation-Based Education in Brazil: Bridging Research and Practice for Healthcare Excellence

**DOI:** 10.31744/einstein_journal/2023EDS3

**Published:** 2023-10-19

**Authors:** Dario Cecilio-Fernandes, Maria Stella Peccin, John Sandars, Thomaz Bittencourt Couto, Alessandra Mazzo

**Affiliations:** 1 Department of Medical Psychology and Psychiatry School of Medical Sciences Universidade Estadual de Campinas Campinas SP Brazil Department of Medical Psychology and Psychiatry, School of Medical Sciences, Universidade Estadual de Campinas, Campinas, SP, Brazil.; 2 Department of Sciences and Human Movement Universidade Federal de São Paulo Santos SP Brazil Department of Sciences and Human Movement, Universidade Federal de São Paulo, Santos, SP, Brazil.; 3 Health Research Institute Edge Hill University Ormskirk England Health Research Institute, Edge Hill University, Ormskirk, England.; 4 Hospital Israelita Albert Einstein São Paulo SP Brazil Hospital Israelita Albert Einstein, São Paulo, SP, Brazil.; 5 Faculdade Israelita de Ciências da Saúde Albert Einstein Hospital Israelita Albert Einstein São Paulo SP Brazil Faculdade Israelita de Ciências da Saúde Albert Einstein, Hospital Israelita Albert Einstein, São Paulo, SP, Brazil.; 6 Faculdade de Odontologia de Bauru Universidade de São Paulo Bauru SP Brazil Faculdade de Odontologia de Bauru, Universidade de São Paulo, Bauru, SP, Brazil.

Simulation-based education (SBE) in health professions education has been increasingly implemented across the world for the past two decades. There has also been a similar trend in Brazil, with SBE being widely implemented in most phases of education, from undergraduate to postgraduate to continued professional development. However, there are still many challenges concerning the effective use of SBE in Brazil, especially due to the low level of country-specific research that can inform its implementation. In this editorial, we will discuss the current challenges of researching SBE to inform best practice in Brazil and also propose a future research agenda to ensure SBE is more effective.

**Figure 1:**
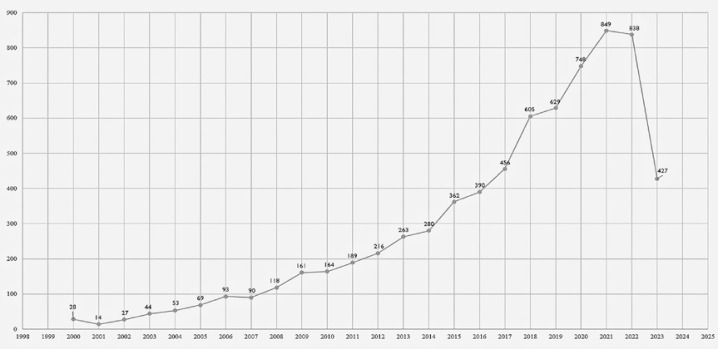
Number of articles published on simulation-based education in Web of Science per year by international authors

**Figure 2:**
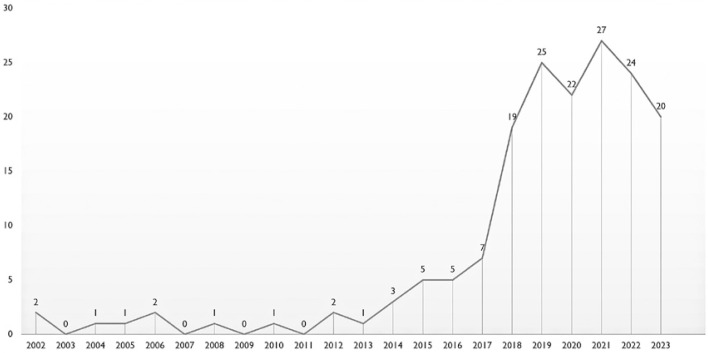
Number of articles published on simulation-based education in Web of Science per year by authors in Brazil

We conducted a search on the Web of Science using terms related to SBE and found a growth in the number of publications related to SBE. We identified 7,113 articles worldwide (Figure 1), but only 168 articles (Figure 2) were by Brazilian authors. We have also noticed that most publications from Brazil are published in Brazilian journals, but not in international journals. One possible explanation is the lack of authors’ proficiency in English and the fact that most publications in high impact journals only have native English speakers as co-authors.^(^^1^^)^ Also, non-native speakers take more time to write and revise in English.^(^^2^^)^ However, this also might be explained by the type of research that has been conducted in Brazil.

The history of SBE in Brazil is recent and the articles identified in the search highlight different periods in the development of research on SBE. First, most of the studies were related to the strategy of SBE, including the discovery and appropriation of the physical, human and material resources. Subsequently, research focuses on distinguishing skills, training, and the development of scenarios and instruments to support and evaluate SBE. Finally, there was an increase in research investigating participants’ satisfaction and whether they had learned after SBE. There were various discussions about the bioethics of training procedures on patients, patient safety policies, and the high mobility of professionals in clinical fields, associated with changes in organizational contexts.

Around 2010, a more in-depth discussion began, especially with the development of scientific events, discussion forums, training of trainers and researchers who looked back at the need for physical, human and material structures, and the need to invest resources that could encourage the use of clinical simulation to be more widely implemented in Brazil. In the same period, the Brazilian Association of Health Simulation (ABRASSIM - *Sociedade Brasileira de Simulação na Saúde*) was founded by a group of health educators and researchers with the purpose of adding professionals who would develop and disseminate SBE. Postgraduate research courses began with the first theses and dissertations focusing on simulation being published. Another milestone was the inclusion of SBE in the Curricular Guidelines for Medicine Courses (2014) and Nursing Courses (2018), which highlighted active learning methods, increasing health courses investments in physical and material resources, with a focus on simulation laboratories.

Despite the recent interest in research, many Brazilian studies are still investigating whether participants enjoy simulation training or whether they learned after simulation training without any further comparison. This means that most of the research focuses on the first two levels of the Kirkpatrick model, which has little value for international journals as it is well established that participants enjoy and learn from simulation training. The Kirkpatrick model has been widely used to classify outcome measurements in health professions education. This model has four levels. The first level is reaction. It measures whether participants are satisfied with the training. The second level is learning. It measures the degree of the intended learning objective acquired by participants. The third level is behaviour. It measures whether participants apply what they learned in practice. Finally, the fourth level is results. It measures whether there was a change in the target outcome.^(^^3^^)^

Another important aspect is that most evidence available to educators include findings from research conducted outside Brazil. This limits the potential application of research to inform local best practices as there often is a lack of information on how the intervention was performed and the barriers encountered.^(^^4^^,^^5^^)^ Without that basic information, the implementation of new strategies is unrealistic and there are recent calls for greater transparency in reporting.^(^^5^^,^^6^^)^ Implementation science has a focus on the implementation of knowledge into practice and it has been widely used in healthcare.^(^^7^^)^ A variety of methods can be combined to understand how evidence can be implemented in practice by identifying factors that are enablers and barriers to changing current practice. It also requires an understanding from individual, organizational, and wider system levels.

There is a vast literature suggesting a decay in both knowledge and skills after SBE, even within a few days of training.^(^^8^^,^^9^^)^ Designing interventions based on cognitive science principles, such as spacing effect and testing effect, is essential for effective SBE, especially to minimise knowledge and skill decay. However, there are few studies comparing the advantages of different cognitive strategies.^(^^10^^)^

We propose a future research agenda addressing the challenges of implementing effective SBE in Brazil. Our key recommendations to advance SBE research include measuring outcomes at the higher third and fourth levels of the Kirkpatrick model, using insights from implementation science in order to understand and overcome the barriers to local implementation, and conducting experimental research that will compare different simulation strategies.
